# Transparency improves the accuracy of automation use, but automation confidence information does not

**DOI:** 10.1186/s41235-024-00599-x

**Published:** 2024-10-08

**Authors:** Monica Tatasciore, Luke Strickland, Shayne Loft

**Affiliations:** 1 The University of Western Australia, 35 Stirling Highway, Perth, WA 6009 Australia; 2 Curtin University, Perth, Australia

**Keywords:** Automation and human cognition, Automation transparency, Automation reliability, Uninhabited vehicle control, Decision-support systems, Automation confidence

## Abstract

Increased automation transparency can improve the accuracy of automation use but can lead to increased bias towards agreeing with advice. Information about the automation’s confidence in its advice may also increase the predictability of automation errors. We examined the effects of providing automation transparency, automation confidence information, and their potential interacting effect on the accuracy of automation use and other outcomes. An uninhabited vehicle (UV) management task was completed where participants selected the optimal UV to complete missions. Low or high automation transparency was provided, and participants agreed/disagreed with automated advice on each mission. We manipulated between participants whether automated advice was accompanied by confidence information. This information indicated on each trial whether automation was “somewhat” or “highly” confident in its advice. Higher transparency improved the accuracy of automation use, led to faster decisions, lower perceived workload, and increased trust and perceived usability. Providing participant automation confidence information, as compared with not, did not have an overall impact on any outcome variable and did not interact with transparency. Despite no benefit, participants who were provided confidence information did use it. For trials where lower compared to higher confidence information was presented, hit rates decreased, correct rejection rates increased, decision times slowed, and perceived workload increased, all suggestive of decreased reliance on automated advice. Such trial-by-trial shifts in automation use bias and other outcomes were not moderated by transparency. These findings can potentially inform the design of automated decision-support systems that are more understandable by humans in order to optimise human-automation interaction.

## Significance statement

Automation is increasingly prevalent in modern workplace settings such as defence, aviation, and healthcare, and often human operators work with automation that assists them to make decisions. Such decision-support systems typically improve decision-making, but instances of inappropriate reliance on automation can lead to the misuse (accepting incorrect advice) or disuse (rejecting correct advice) of advice which can result in serious consequences in safety-critical work settings. This study manipulated two work-design factors that can potentially enhance the understandability and predictability of automated advice; increased automation transparency regarding the reasoning underlying advice, and the provision of automation confidence information (i.e., automation’s prediction of its accuracy on a case-by-case basis). Increased transparency resulted in more accurate automation use, faster correct decisions, lower perceived workload, and increased perceived trust and usability. Automation confidence information had no net impact on outcome variables, but there was decreased automation reliance on low compared to high confidence trials (indicated by increased correct rejection rates, decreased hit rates, slowed decisions, and increased perceived workload), which was not moderated by increased transparency. These outcomes have potential application to inform the design of more predictable automated decision-support systems to optimise human-automation interaction.

## Introduction

Automation is increasingly prevalent in modern workplaces. For example, people work with decision-support systems that provide information, recommendations, and/or predictions to enhance decision-making (NASEM, 2022). Decision-support systems are common across work settings such as defence (e.g., advice for deploying uninhabited vehicles), aviation, healthcare, manufacturing, cybersecurity, and process control. Despite the potential for decision-support to improve decision-making and other outcomes, mis-calibrated trust in automation can result in either over-reliance (*automation bias*; Mosier et al., 1996; Parasuraman & Manzey, 2010) and the *misuse* of automated advice (accepting incorrect advice), or under-reliance and the *disuse* of advice (rejecting correct advice; Lee & See, 2004; Parasuraman & Riley, 1997).

Trust in, and reliance on, automation is influenced by numerous factors, including operator characteristics (e.g., predisposition to trust), contextual factors (e.g., task complexity), and automation characteristics (e.g., reliability; Hoff & Bashir, 2015). Reliance can be impacted by factors other than trust (e.g., decision risk, manual ability; Patton & Wickens, 2024), but nonetheless calibrated trust is critical for correctly relying on automated advice when it is accurate and for rejecting inaccurate advice (Lee & See, 2004). Trust calibration reflects human expertise in predicting automation errors, based upon understanding how automation performs under various conditions (Carter et al., 2024; Strickland et al., 2024), and thus work designs that improve understanding of automation performance could facilitate automation use. Indeed, increasing *transparency* regarding the rationale underlying automated advice can improve the accuracy of automation use (see reviews by Bhaskara et al., 2020; Van de Merwe et al., 2022). Providing automation *confidence* information (i.e., automation’s expected accuracy) also increases the predictability of automation errors, and thus may improve automation use (e.g., McGuirl & Sarter, 2006).

To predict automation trust and reliance in workplace settings, it is essential to not only establish which factors enhance the understandability and predictability of automation errors, but also how those factors potentially interact. It is plausible that providing automation transparency and confidence information could have interacting effects on automation use. For example, low confidence signalling by automation may encourage greater scrutiny of transparency information at key moments when automation is more likely to error, which should be more beneficial when high compared to low transparency is being provided. We examined the effects of transparency, confidence information, and their potential interaction on the accuracy of automation use and other outcome variables.

### Automation transparency

There are many definitions in the literature of automation transparency as a work design principle. For example, automation transparency has been defined as providing “a real-time understanding of the actions of the AI system” (NASEM, 2022, p.31), or enhancing the “understandability and predictability of a system” (Endsley et al., 2003, p.146). The Situation-Awareness Agent-Based Transparency (SAT) model (Chen et al., 2014) proposes three tiers of transparency design including the goals and intent of the automation (Level 1), rationale underlying advice (Level 1 + 2), and projected outcomes if advice is actioned (Level 1 + 2 + 3). Whilst the SAT model is prominent in the literature, the task domain in which automation is being applied influences how transparency is operationalised and designed (Skraaning & Jamieson, 2021; Van de Merwe et al., 2022, 2024). Narrative reviews and meta-analyses indicate that increased transparency can be associated with more accurate automation use, without costs to decision time or perceived workload (for reviews see Bhaskara et al., 2020; Sargent et al., 2023; Van de Merwe et al., 2022).

Numerous studies have applied uninhabited vehicle (UV) management tasks to examine the effects of increased transparency, and this is the task under investigation here due to its relevance to Defence. Increased transparency in UV management has resulted in more accurate automation use, without costs to decision time or perceived workload (Gegoff et al., 2023; Mercado et al., 2016; Stowers et al., 2020; Tatasciore & Loft, 2024; Tatasciore et al., 2023). Additionally, on occasions increased transparency has led to higher trust and usability ratings (Mercado et al., 2016; Stowers et al., 2020; Tatasciore & Loft, 2024).

However, Tatasciore et al. (2023) and Tatasciore and Loft (2024) found that increased transparency resulted in a bias towards agreeing with automated advice. Despite increased sensitivity (i.e., better discrimination between correct and incorrect advice) with high transparency, these benefits were more due to improved hit rates (accepting correct advice) rather than improved correct rejection rates (rejecting incorrect advice). Further, Bhaskara et al. (2021) found that increased transparency led to increased automation bias, lower correct rejection rates, and *poorer* sensitivity. Thus, humans may not always process high transparency information adequately to validate automated advice. Our first aim was to replicate the benefit of increased transparency reported by Gegoff et al. (2023), Tatasciore et al. (2023), and Tatasciore and Loft (2024) on the accuracy of automation use.

In addition to increased transparency, it may be useful to provide humans with feedback from automation regarding its limitations. Specifically, humans may benefit from confidence information regarding automated advice (i.e., automation’s prediction of its accuracy), presented alongside transparency information on a case-by-case (i.e., trial-by-trial) basis (Mosier & Manzey, 2019). The provision of confidence alongside increased transparency could work together to calibrate trust in automated advice.

### Automation confidence

Theories of supervisory monitoring (Moray & Inagaki, 2000; Senders, 1983) and attentional control (Steelman et al., 2011; Wickens et al., 2015) propose that the likelihood of attending to information, and subsequent depth of information processing, is affected by the perceived expected value of that information in relation to task goals (i.e., people seek out information that they believe has higher expected value). According to supervisory monitoring theories then, confidence information should change the expected value of verifying task information (e.g., transparency information). Arguably, it is rational for humans to rely more on highly confident automated advice (Moray, 2003). Conversely, when automation indicates lower confidence (i.e., indicating a higher probability that its advice is incorrect), it should encourage a higher expected value for attending to and scrutinising task information in order to check for automated advice errors.

Conceptually, automation confidence information overlaps with SAT Level 3 transparency (projected outcomes). However, confidence information is specifically focused on automation’s statistical uncertainty, rather than insight into its inner workings. Such probability information is known to be subject to a range of human biases (Tversky & Kahneman, 1992; Zhang & Maloney, 2012), which vary with task context (Wulff et al., 2018). Thus, for our purposes, it is useful to treat confidence as conceptually distinct from transparency, and confidence information can be presented alongside low and high transparency. However, transparency aside, there are relatively limited, and mixed, findings regarding how individuals perceive automation confidence and subsequent effects on automation use.

McGuirl and Sarter (2006) examined how automation confidence impacted pilot in-flight icing decisions. Confidence was presented in a graph display and updated on a case-by-case basis allowing pilots to assess current automation confidence (high, variable, or low). Pilots receiving confidence as opposed to overall reliability information were less prone to automation bias. However, a common strategy reported by pilots was to invariably disagree with low confidence advice. This was problematic when the automation reported low confidence but was still correct in its advice (i.e., automation disuse).

Positive outcomes have also been observed with graded likelihood alarm systems (e.g., “ok”, “potentially too high”, “too high”) compared to binary alarm systems (e.g., “ok”, “too high”), presumably because graded information improved estimation of the likelihood of critical events during alerts and thus graded advice whether to follow the alert (Wiczorek & Manzey, 2014; Wiczorek et al., 2014).

However, other studies have found no benefit of graded aid certainty. Bartlett and McCarley (2017, 2019) examined performance on an aided task where participants classified dot images as blue or orange dominant. The aid provided an estimate of signal strength via a numeric rating (Bartlett & McCarley, 2017), likelihood ratio (e.g., 39:1), or percentage confidence rating (Bartlett & McCarley, 2019). Graded aid certainty (regardless of type) did not improve automation use (also see Endsley & Kiris, 1994).

Given these mixed findings, further research is required to examine whether presenting automation confidence can improve the accuracy of automation use. The current study examined the impact of categorical confidence (i.e., somewhat or highly confident) on the accuracy of automation use.

### Automation transparency and confidence information

We were also interested in the potential interactive effects between transparency and confidence. To our knowledge, no prior research has tested for interactions between transparency and confidence, where confidence is provided on a trial-by-trial basis. A recent study demonstrated that transparency can interact with “automation reliability” knowledge, where participants were informed about reliability *prior* to blocks of trials (Gegoff et al., 2023). Higher transparency mitigated the disuse associated with lower compared to higher reliability automation. Further, higher transparency improved the correct rejection of advice in low-reliability blocks. However, high transparency did not alleviate the misuse (i.e., lower correct rejection) linked with high-reliability automation use. Gegoff et al. concluded that participants may not have examined high transparency information closely enough to validate advice when using high-reliability automation (Moray, 2003; Wickens et al., 2015), and thus it is possible they may do the same in the current study on high confidence trials.

Relatedly, some studies have examined the impact of presenting information relating to perceptions of uncertainty in the task environment (e.g., “it is uncertain how fog will affect UV speed”) along with high transparency (Mercado et al., 2016; Stowers et al., 2020). Information about environmental uncertainty, alongside high transparency, can be beneficial (e.g., increased hit rates). Environmental uncertainty information and confidence information are related in that both provide an indication of the likelihood that the automation is correct. However, the concepts should be distinguished. Although environmental uncertainty information instils doubt about environmental factors that the automation incorporates (inputs), it does not provide probability values surrounding its advice/output accuracy, which may be affected by complex interactions of multiple environmental inputs. In the current study, confidence information provides a direct categorical statement about how confident the automation is overall in its final advice (i.e., estimated certainty/reliability). The distinction is important because environmental uncertainty and confidence information may have different impacts on the cognitive processes involved in using automated advice. For example, when environmental uncertainty information is provided it is still necessary to determine the level of transparency information scrutiny required to decide if advice can be relied on. In contrast, automation confidence signalling (i.e., low automation confidence signalling) may serve as a cue to direct attention towards transparency information at the most appropriate times (i.e., when automation is more likely to error), and if so high transparency information should then be more beneficial than low transparency in aiding humans with subsequently understanding the rationale underlying automated advice and in predicting the reliability of that advice.

The current study examined whether the effects of transparency and confidence interact. Because transparency and confidence each affect the predictability of automation errors (Carter et al., 2024; Lee & See, 2004), providing both could have redundant effects on outcomes. For example, operators might attend to confidence instead of transparency information or vice versa, if one type of information suffices to reasonably predict automation accuracy. Alternatively, either factor could amplify the effects of the other. As previously mentioned, increased transparency may assist with the verification and use of variable automation confidence information by facilitating understanding of the rationale underlying advice on a case-by-case basis.

### Current study

The UV management task used in the current study required participants to choose the optimal UV to complete missions by assessing UV capabilities (e.g., fuel consumption), weightings of capabilities, and environmental factors affecting capabilities. The *Recommender* (decision aid) advised the optimal UV after considering these factors. The recommender was 75% reliable, requiring participants to either agree with the advice and select Plan A, or to choose Plan B. This reliability exceeds the 70% reliability threshold which increases the probability that humans rely on automation (Wickens & Dixon, 2007), while providing enough data points for statistical power.

A mixed design was used, with confidence information (present, absent) the between-subjects factor, and transparency (low, high) the within-subjects factor. The SAT model was used as a guiding model to design low versus high transparency conditions as this is a commonly used model in military task applications, but the transparency designs were also informed via consultation with Australian Defence. The low transparency condition broadly equated to SAT Level 1 (e.g., information about the automation’s goals/intent) providing information about how the automation assessed the importance of UV capabilities. The high transparency condition broadly equated to SAT Level 1 + 2 + 3 by providing further information about *how* the automation made its calculations (i.e., SAT 2), including which environmental factors it considered and the *projected impact* of these factors on the two UV plans (i.e., SAT Level 3).

The current study aimed to replicate the benefits of increased transparency on the accuracy of automation use previously reported in UV management. More accurate automation use (i.e., higher hit rates and sensitivity), without costs to decision time or perceived workload, was expected with increased transparency. Increased transparency may also increase perceived trust and usability.

We also examined the impact of presenting categorical confidence information on a trial-by-trial basis on the accuracy of automation use. Lower compared to higher confidence signalling may improve correct rejection rates due to greater scrutiny of automated advice. However, lower confidence may also lead participants to be more likely to disagree with correct advice (McGuirl & Sarter, 2006), lowering hit rates compared to higher confidence trials. Conversely, hit rates may improve on higher compared to lower confidence trials, but at a cost to the correct rejection of advice when it is (rarely) incorrect. If this is the case, there may be no net benefit to sensitivity (i.e., discrimination between correct and incorrect advice) from the provision of confidence information. It also is unclear how confidence will impact decision time, perceived workload, or perceived trust or usability, but one might expect slower decision times and increased perceived workload with decreased automation reliance.

The final question concerned whether increased transparency and confidence would interact to further improve the accuracy of automation use. As discussed, if lower confidence serves as a higher expected value cue to direct attention to transparency information, we would expect more correct rejections, *and* higher hit rates, on low confidence trials for high compared to low transparency conditions. High transparency may also protect against the likely negative effect of higher confidence signalling on the correct rejection of automated advice. If these outcomes are observed, the benefit of increased transparency on the accuracy of automation use will be greater when automation confidence information is also provided.

## Methods

### Participants

Participants included 131 (91 female, 38 male, 2 non-binary; *M* = 20.01 years) undergraduate students who were provided course credit and a maximum performance incentive of AUD$30. Participants were randomly allocated to either the confidence information present (*N* = 67) or absent (*N* = 64) conditions.

### Uninhabited vehicle management task

**Fig. 1:**
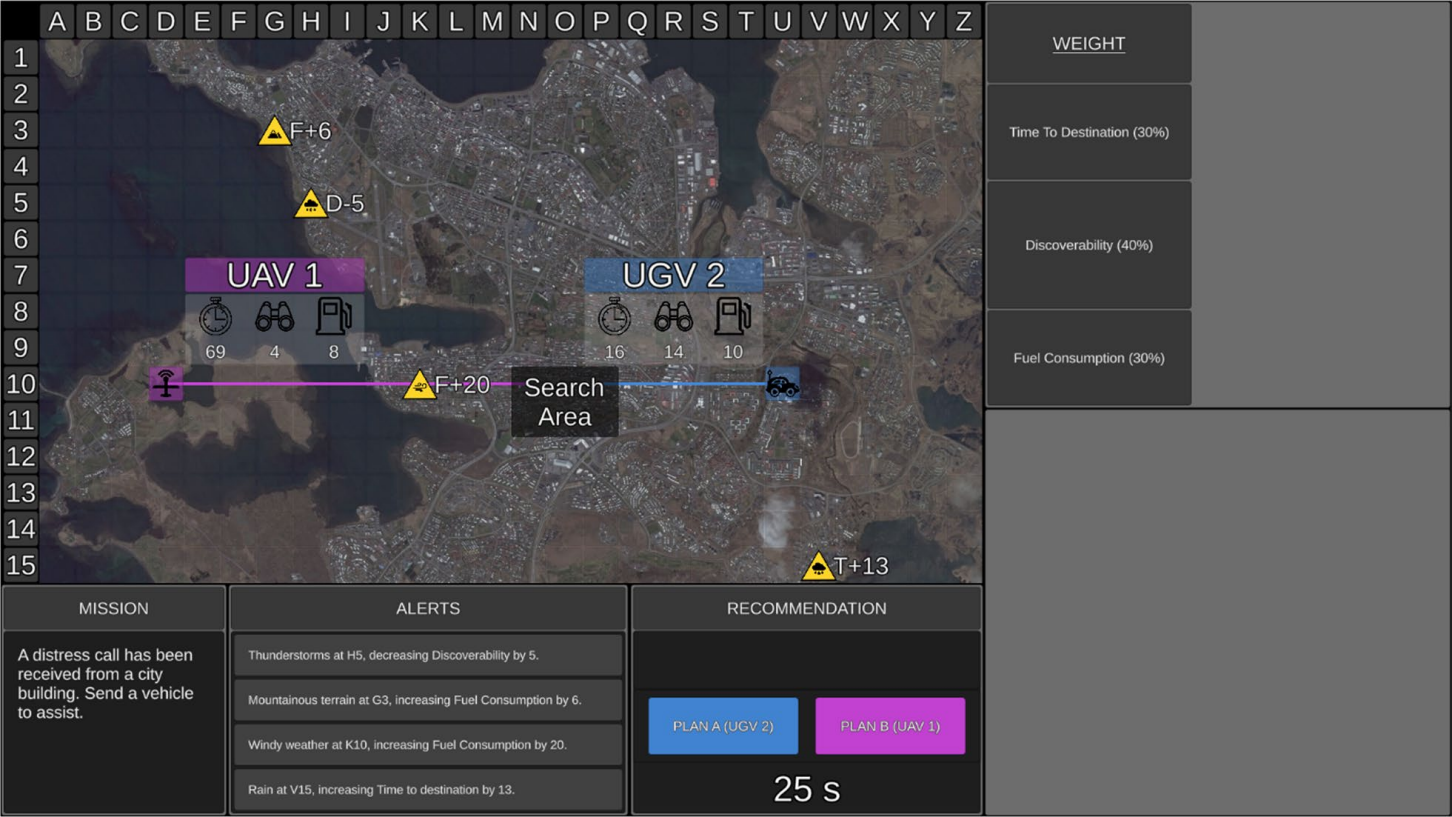
The uninhabited vehicle management task with low transparency. *Note* Here, the search area, UV capabilities, UV path to the search area, and environmental factor symbols are presented on the urban tactical map. A relevant factor symbol is placed on the path of UAV 1, and the impact of environmental factors can also be seen in the alerts window. The Recommender’s advice is presented in the Recommendation window, along with the time remaining and two UV selection buttons. In this example, the Recommender correctly recommended UGV 2 as Plan A. Low transparency information is presented in the table (top right) display and outlines how the Recommender evaluated the weighting of each capability, with larger rows signifying higher weightings. Confidence information is absent in this example. Adapted from Tatasciore and Loft (2024)

The UV task was presented on a single desktop monitor (Fig. 1). Participants completed 200 trials (missions), with 100 trials completed in the low transparency block and 100 in the high transparency block. Mission statements were displayed in the mission window for face validity, but they did not relate to optimal UV selection. Rural, coastal, or urban areas were presented as an aerial tactical map. The tactical map also presented the search area and two UVs (ground [UGV]; surface [USV]; or aerial [UAV]), which were randomly numbered 1 or 2 and assigned a unique colour (blue or purple). Attached to each UV was a line illustrating its path to the search area.

The optimal UV was selected based on UV capabilities (time to destination, discoverability, fuel consumption), capability weightings, and environmental factors impacting capabilities. Time to destination (value displayed adjacent to timer symbol in Fig. 1) denoted the duration the UV would take to reach the search area (lower = quicker). Discoverability (binocular symbol) denoted how discoverable the UV was by third parties (lower = less discoverable). Fuel consumption (fuel gauge symbol) denoted the amount of fuel the UV would consume to reach the search area (lower = less fuel).

Displayed in the weightings window were importance weightings (%) for each UV capability (Fig. 1), which were pre-set for each mission (not determined by automation). One of five weighting sets was used for each mission, with weighting sets varying in difficulty. Sets were considered hard if two capabilities had equal weightings (e.g., 45%, 45%, 10%), requiring consideration of which UV had lower scores on two capabilities. Easy sets had one highly weighted capability (e.g., 80%, 10%, 10%), making it only necessary to consider which UV scored lower on the highest weighted capability.

During each mission, four environmental factors represented as yellow environmental factor symbols were displayed on the tactical map, which were relevant or irrelevant to UV capabilities. When relevant, environmental factor symbols were positioned along the path of a UV, whereas irrelevant factors were not and could be disregarded (Fig. 1). Environmental factor symbols depicted the type of factor (e.g., rain), capability impacted (*T* = time to destination, *D* = discoverability, *F* = fuel consumption), direction of impact (+ = positive, − = negative), and level of impact. Messages in the alerts window also listed factors and their impact. For each mission, there were one to three relevant factors.

The Recommender advised the optimal UV as Plan A, and the other as Plan B (Recommendation window; Fig. 1), after examining UV capabilities, their weightings, and environmental factors. Participants had 25 s (maximum) to either accept the Recommender’s advice and choose Plan A, or to choose Plan B. If no response was made within this time an incorrect response was recorded, and the next trial was presented. Following each mission, feedback was provided on the accuracy of the Recommender and the participant’s decision.

Participants were aware of the Recommender’s goals and intent, and further, the low transparency information provided in the table display illustrated how the Recommender assessed the importance of UV capabilities, with table rows displayed larger for higher weighted capabilities (Fig. 1). Note that when participants completed manual unaided training trials, the size of table rows was identical for each capability weighting.

**Fig. 2:**
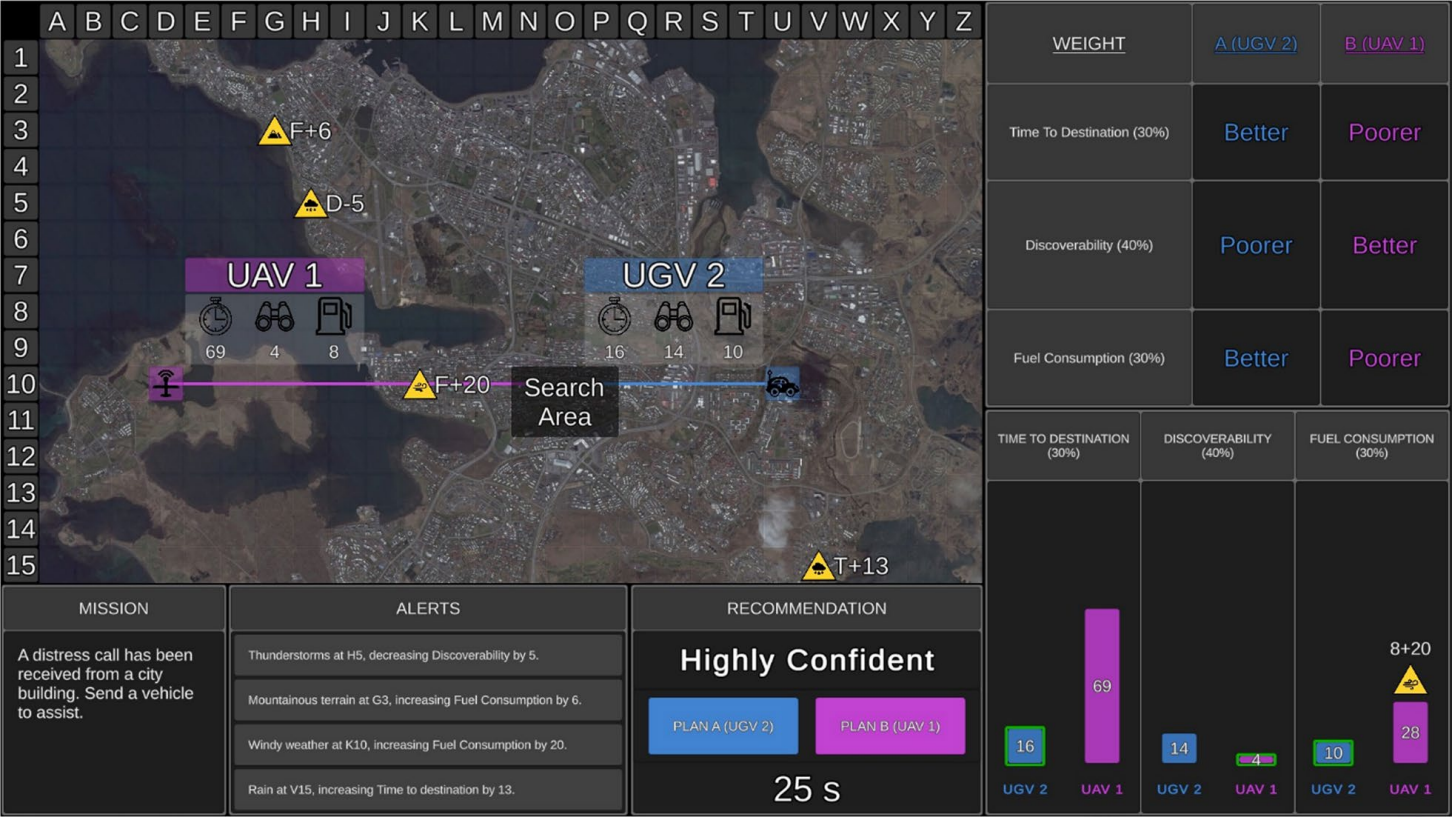
The uninhabited vehicle management task with high transparency and confidence information. *Note* Here the Recommender has reported that it is highly confident in its recommendation. High transparency information is displayed in the table (top right) and graph (bottom right) displays. Plan A is positioned on the left and Plan B on the right in both the table and graph displays. In addition to presenting how the Recommender evaluated the weighting of each UV capability, the table display also presented which UV the Recommender considered to be better and poorer on each capability. The graph display presented how the Recommender calculated the score for each capability. When there were relevant environmental factors, the factor symbol was positioned above the relevant bar on the graph, along with the value added or subtracted from the original score. The Recommender’s calculated score was presented in the bar. Finally, the UV that the Recommender considered to be better on each capability was outlined in green. Adapted from Tatasciore and Loft (2024)

When high transparency information was provided, the table display also indicated which UV the Recommender considered to be “better” and “poorer” on each capability (Fig. 2). A graph display was also provided with high transparency. This display comprised of three bar graphs illustrating how the Recommender calculated the score for each capability, and which factors it considered. When an environmental factor was relevant to a UV capability, the factor symbol was presented above the relevant bar. Above the factor symbol was the factor impact value that the Recommender added or subtracted from the original capability score. The final calculated score was depicted within the respective bar (shorter bars = better capability). High transparency therefore presented information about the automation’s projection of the consequences of variability in the task environment, and thus projected outcomes if its advice was actioned. A green border was placed around the bar of the UV that the Recommender considered to be better on each capability. In each graph, Plan A was always positioned on the left-hand side.

The reliability of the Recommender was 75% (it advised the incorrect UV as Plan A on 25% of trials). Participants were trained that “*The Recommender is only reasonably reliable automation, and as such it may not always recommend the optimal plan*.” When the Recommender’s advice was incorrect it either: missed a relevant factor, miscalculated the impact of a relevant factor, or a combination of these errors across different relevant factors.

With high transparency, when the Recommender missed a relevant environmental factor, the factor symbol would be missing from above the relevant bar in the graph display indicating it was missed by the Recommender, and the original score incorrectly presented. When the Recommender considered but miscalculated the impact of a relevant factor, the factor symbol would be presented on the graph, but the value on the graph that the Recommender added or subtracted was incorrect, leading to an incorrect final score. Additionally, the green border may have been placed around the incorrect UVs bar on the graph. Due to these errors, the UV that the Recommender considered to be “better” and “poorer” on each UV capability in the table display could also be incorrect.

For 10% of the reliable (i.e., correct automated advice) trials, despite advising the correct UV as Plan A, the Recommender still made one or more of the above errors, but they did not impact in terms of their direction or magnitude to make the Recommender’s advice incorrect. Such trials were incorporated to prevent participants from selecting Plan B upon detecting any error made by the Recommender in the graph display (Gegoff et al., 2023; Tatasciore et al., 2023).

Confidence information was presented on a trial-by-trial basis in the Recommendation Window (Fig. 2) and referred to how confident the Recommender was in its advice. The Recommender was either “highly” or “somewhat” confident. Although studies have found similarities in human judgement accuracy across linguistic and numeric representations of probability values (e.g., Bisantz et al., 2005), we used categorical representations of confidence to minimise the cognitive effort required to interpret numerical estimates (Bhatt et al., 2021). However, one drawback of using predefined categories is that users may not be aware of or misinterpret the threshold criteria of the categories (Bhatt et al., 2021). As such, participants were trained that when the Recommender was highly confident it was 90% confident, and when it was somewhat confident it was 60% confident.

The 200 trials were split into two 100-trial blocks. In each block, there was a similar number of capability weighting sets, relevant environmental factors, factor impacts, and type of Recommender errors. The presentation order of blocks and transparency was counterbalanced. The order of unique trials was randomised and yoked such that sets of four participants received the same randomised order of trials, with only two of the four participants receiving confidence information. Table 1 presents the number of trials assigned somewhat confident versus highly confident information, split by whether the advised plan was correct/incorrect. The assignment of confidence to specific mission trials (i.e., correct vs. incorrect automated advice) was randomised in accordance with the breakdown in Table 1.

**Table 1:** Breakdown of confidence information across trials

Information	Recommended plan correct (Plan A correct)	Recommended plan incorrect (Plan B correct)
Somewhat confident	30	20
Highly confident	45	5

### Measures

#### Automation use accuracy

Hit rate represented the proportion of trials that Plan A was correctly selected. Correct rejection rate represented the proportion of trials that Plan B was correctly selected when the advised Plan A was incorrect. The Signal Detection metric *d′* evaluated sensitivity to discriminate Plan A from B. The metric *c* evaluated bias towards agreeing with automated advice (negative values signify greater bias). Extreme hit or false alarm values (0 or 1) were adjusted by replacing values of 0 with 0.5/*n*, and values of 1 with (*n* − 0.5)/*n*, where n is the number of signal (Plan A correct) or noise (Plan B correct) trials (Macmillan & Kaplan, 1985).

#### Decision time

Decision times were based on correct decisions only.

#### Workload

After each mission, the Air Traffic Workload Input Technique (ATWIT; Stein, 1985) was used to measure perceived workload. Participants rated their workload from 1 (very low) to 10 (very high) within 5 s.

#### Trust

A six-item questionnaire adapted from Merritt (2011) measured trust. A 5-point Likert scale from 1 (strongly disagree) to 5 (strongly agree) was used to rate items.

#### System usability scale

We adapted the 10-item System Usability Scale (SUS; Brooke, 1996) to measure perceived usability. A 5-point Likert scale from 1 (strongly disagree) to 5 (strongly agree) was used to rate items. Some items were reverse-scored and then all items were added and multiplied by 2.5 to yield a score from 0 to 100.

### Procedure

The total duration was 2 h. Training started with a 20 min audiovisual PowerPoint detailing the process for manually completing missions, followed by 20 manual practice trials. Participants then viewed a PowerPoint presentation specific to the transparency assigned during the first block. Training regarding confidence information was also included in this presentation for participant's assigned confidence. Participants then completed the first block of 100 trials with a 1 min (minimum) break after 50 trials. Participants then watched a PowerPoint specific to the transparency assigned during the second block, after which they completed 100 trials (1 min minimum break after 50 trials). Participants completed the trust and SUS questionnaires after each block (counterbalanced order).

## Results

Descriptive statistics for manual training trials as a function of confidence condition are presented in Table 2, with no significant differences on outcome variables (smallest *p* = 0.39).

**Table 2:** Descriptive statistics for manual training trials as a function of confidence condition

	Automation confidence	
	Absent	Present
UV selection accuracy	.84 (.13)	.82 (.12)
Decision time (s)	14.02 (2.83)	14.40 (2.13)
Perceived workload	4.16 (1.31)	4.15 (1.42)

s, seconds

### Automation transparency and confidence

**Fig. 3:**
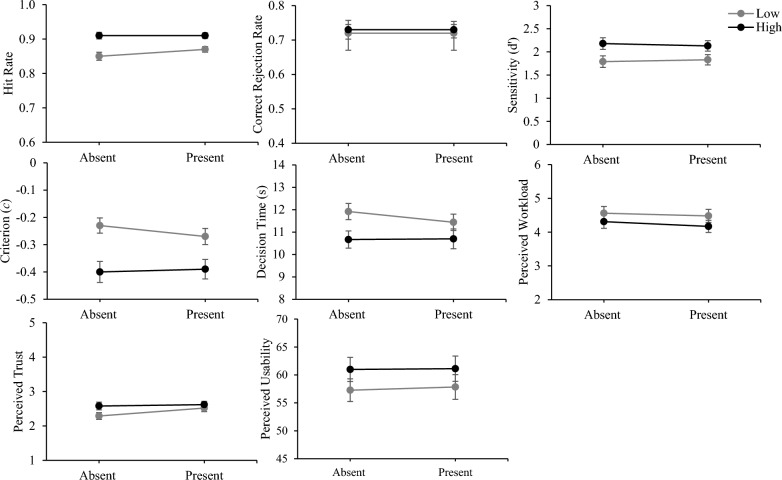
Accuracy of automation use, decision time, perceived workload, trust, and usability as a function of automation transparency and confidence. *Note* Error bars represent the mean plus or minus the standard error. Low = low transparency; High = high transparency

Table 3 presents marginal means for transparency and confidence for each outcome variable. Descriptive statistics for outcome variables as a function of transparency and confidence are presented in Table 4 (also see Fig. 3). A series of 2 Transparency (low, high) × 2 Confidence (present, absent) mixed ANOVAs were conducted. Main effects of transparency, or interactions between transparency and confidence, would be followed with planned contrasts comparing low to high transparency when confidence was absent and present. Partial eta squared (small = 0.01, medium = 0.06, large = 0.14) was used to estimate effect sizes for *F*-tests, and Cohen’s *d* (small = 0.20, medium = 0.50, large = 0.80) for *t*-tests (Cohen, 1992).

**Table 3:** Marginal means for transparency and confidence for each outcome variable

	Hit	CR	*d′*	*c*	DT	Workload	Trust	Usability
*Transparency*								
Low T	.91 (.07)	.72 (.20)	1.81 (.96)	− .25 (.23)	11.67 (2.94)	4.52 (1.60)	2.41 (.83)	57.56 (17.15)
High T	.86 (.08)	.73 (.21)	2.15 (.96)	− .39 (.30)	10.68 (3.34)	4.24 (1.52)	2.60 (.84)	61.05 (17.88)
*Confidence*								
Absent	.88 (.08)	.73 (.19)	1.99 (.91)	− .31 (.22)	11.29 (2.55)	4.44 (1.53)	2.44 (.73)	59.12 (14.62)
Present	.89 (.06)	.72 (.19)	1.98 (.84)	− .33 (.21)	11.07 (2.83)	4.32 (1.44)	2.57 (.70)	59.48 (16.79)

Standard deviations are presented in parentheses

Low T, low transparency; High T, high transparency; CR, correct reject; *d’*, sensitivity; *c*, criterion (response bias); DT, correct decision time in seconds

**Table 4:** Descriptive statistics for test trials as a function of transparency (low, high) and confidence (present, absent)

	Hit	CR	*d′*	*c*	DT	Workload	Trust	Usability
*Confidence absent*								
Low T	.85 (.09)	.72 (.20)	1.79 (1.00)	− .23 (.22)	11.92 (2.88)	4.56 (1.61)	2.29 (.80)	57.27 (16.30)
High T	.91 (.08)	.73 (.22)	2.18 (1.01)	− .40 (.31)	10.67 (3.08)	4.31 (1.58)	2.58 (.90)	60.98 (17.30)
*Confidence present*								
Low T	.87 (.07)	.72 (.21)	1.83 (.92)	− .27 (.24)	11.44 (2.99)	4.48 (1.59)	2.52 (.84)	57.84 (18.03)
High T	.91 (.07)	.73 (20)	2.13 (.93)	− .39 (.29)	10.70 (3.60)	4.17 (1.47)	2.62 (.78)	61.12 (18.54)

Standard deviations are presented in parentheses

Low T, low transparency; High T, high transparency; CR, correct reject; *d’*, sensitivity; *c*, criterion (response bias); DT, correct decision time in seconds

### Automation use accuracy

We found a main effect of transparency on hit rates, *F*(1,129) = 65.28, *p* < 0.001, ${\eta }_{\rho }^{2}$=0.34, with higher hit rates with high compared to low transparency. There was no main effect of confidence, *F* < 1. There was a marginal interaction between transparency and confidence, *F*(1,129) = 3.98, *p* = 0.05, ${\eta }_{\rho }^{2}$=0.03. When confidence was both absent, *t*(63) = 6.61, *p* < 0.001, *d* = 0.70, and present, *t*(66) = 4.67, *p* < 0.001, *d* = 0.57, hit rates were higher with high compared to low transparency.

For correct rejection rates, there were no main effects of transparency, *F* < 1, or confidence, *F* < 1, and no interaction, *F* < 1.

There was a main effect of transparency on sensitivity (*d′*), *F*(1,129) = 24.53, *p* < 0.001, ${\eta }_{\rho }^{2}$=0.16, with greater sensitivity with high compared to low transparency. There was no main effect of confidence, *F* < 1, and no interaction, *F* < 1. When confidence was both absent, *t*(63) = 3.72, *p* < 0.001, *d* = 0.39, and present, *t*(66) = 3.25, *p* = 0.002, *d* = 0.32, sensitivity was greater with high compared to low transparency.

For response bias (*c*), there was a main effect of transparency, *F*(1,129) = 28.62, *p* < 0.001, ${\eta }_{\rho }^{2}$=0.18, with greater bias towards agreeing with the automation with high compared to low transparency. There was no main effect of confidence, *F* < 1, and no interaction, *F*(1,129) = 1.04, *p* = 0.31. When confidence was both absent, *t*(63) = 4.65, *p* < 0.001, *d* = 0.63, and present, *t*(66) = 2.98, *p* = 0.004, *d* = 0.45, participants were more biased towards agreeing with automation with high compared to low transparency.

### Correct decision time

There was a main effect of transparency, *F*(1,129) = 12.11, *p* < 0.001, ${\eta }_{\rho }^{2}$=0.09, with faster decision times with high compared to low transparency. There was no main effect of confidence, *F* < 1, and no interaction, *F* < 1. When confidence was absent, decision times were faster with high compared to low transparency, *t*(63) = 3.22, *p* = 0.002, *d* = 0.42. However, when confidence was present, there was no difference in decision times between low and high transparency conditions, *t*(66) = 1.77, p = 0.08.

### Workload

We found a main effect of transparency, *F*(1,129) = 10.13, *p* = 0.002, ${\eta }_{\rho }^{2}$=0.07, with lower perceived workload with high compared to low transparency. There was no main effect of confidence, *F* < 1, and no interaction, *F* < 1. When confidence was both absent, *t*(63) = 2.17, *p* = 0.03, *d* = 0.16, and present, *t*(66) = 2.34, *p* = 0.02, *d* = 0.20, perceived workload was lower with high compared to low transparency.

### Trust

We found a main effect of transparency, *F*(1,129) = 7.15, *p* = 0.01, ${\eta }_{\rho }^{2}$=0.05, with higher trust ratings with high compared to low transparency. There was no main effect of confidence, *F*(1,129) = 1.11, *p* = 0.30, and no interaction, *F*(1,129) = 1.64, *p* = 0.20. When confidence was absent, trust ratings were higher with high compared to low transparency, *t*(63) = 2.68, *p* = 0.01, *d* = 0.34. However, when confidence was present, there was no difference in trust ratings between low and high transparency conditions, *t*(63) = 1.03, *p* = 0.31.

### Usability

For usability, there was a main effect of transparency, *F*(1,129) = 6.61, *p* = 0.01, ${\eta }_{\rho }^{2}$=0.05, with higher usability ratings with high compared to low transparency. There was no main effect of confidence, *F* < 1, and no interaction, *F* < 1. When confidence was both absent, *t*(63) = 1.79, *p* = 0.08, and present, *t*(63) = 1.85, *p* = 0.07, the difference in usability ratings between low and high transparency conditions did not reach significance.

### Automation transparency (confidence present only)

**Fig. 4:**
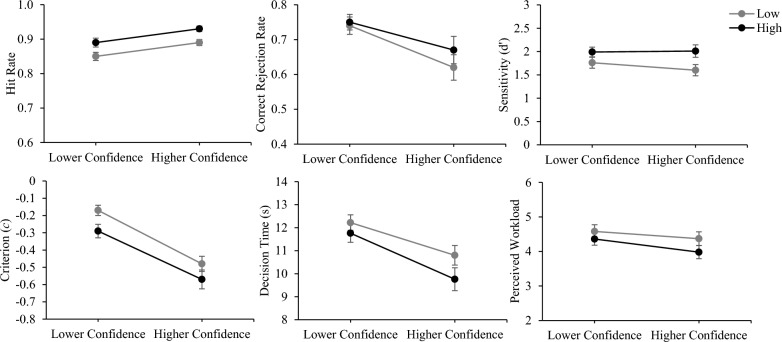
Accuracy of automation use, decision time, and perceived workload as a function of automation transparency and level of confidence. *Note* Error bars represent the mean plus or minus the standard error. Low = low transparency; High = high transparency

Table 5 presents marginal means for transparency and confidence for the condition in which confidence information was presented. Descriptive statistics for the condition in which confidence information was presented, as a function of transparency and confidence are presented in Table 6 (also see Fig. 4). We ran a series of 2 Transparency (low, high) × 2 Confidence (lower, higher) repeated measures ANOVAs. Main effects of confidence were followed with planned contrasts comparing lower to higher confidence trials at each level of transparency.

**Table 5:** Marginal means for transparency and confidence for the condition in which confidence was presented

	Hit	CR	*d′*	*c*	DT	Workload
*Transparency*						
Low T	.87 (.07)	.72 (.21)	1.83 (.92)	− .27 (.24)	11.44 (2.99)	4.48 (1.59)
High T	.91 (.07)	.73 (.20)	2.13 (.93)	− .39 (.29)	10.70 (3.60)	4.17 (1.47)
*Confidence*						
Lower confidence	.87 (.08)	.74 (.17)	1.87 (.81)	− .23 (.21)	11.99 (2.49)	4.47 (1.44)
Higher confidence	.91 (.06)	.64 (.28)	1.80 (.94)	− .53 (.34)	10.28 (3.27)	4.18 (1.48)

Standard deviations are presented in parentheses

Low T, low transparency; High T, high transparency; CR, correct reject; *d’*, sensitivity; *c*, criterion (response bias); DT, correct decision time in seconds

**Table 6:** Descriptive statistics for the condition in which confidence was presented, as a function of transparency and level of confidence (lower or higher)

	Hit	CR	*d′*	*c*	DT	Workload
*Low T*						
Lower confidence	.85 (.10)	.74 (.20)	1.76 (.96)	− .17 (.24)	12.22 (2.75)	4.58 (1.58)
Higher confidence	.89 (.07)	.62 (.30)	1.60 (.98)	− .48 (.36)	11.76 (3.24)	4.37 (1.62)
*High T*						
Lower confidence	.89 (.10)	.75 (.18)	1.99 (.85)	− .29 (.32)	10.80 (3.50)	4.36 (1.46)
Higher confidence	.93 (.07)	.67 (.32)	2.01 (1.09)	− .57 (.45)	9.76 (4.07)	3.98 (1.56)

Standard deviations are presented in parentheses

Low T, low transparency; High T, high transparency; CR, correct reject; *d’*, sensitivity; *c*, criterion (response bias); DT, correct decision time in seconds

### Automation use accuracy

We found a main effect of transparency, *F*(1,66) = 23.88, *p* < 0.001, ${\eta }_{\rho }^{2}$=0.27, and confidence, *F*(1,66) = 19.66, *p* < 0.001, ${\eta }_{\rho }^{2}$=0.23, with higher hit rates with higher compared to lower confidence. There was no interaction, *F* < 1. With low, *t*(66) = 4.14, *p* < 0.001, *d* = 0.46, and high transparency, *t*(66) = 3.48, *p* < 0.001, *d* = 0.46, hit rates increased with higher compared to lower confidence.

We found a main effect of confidence on correct rejection rates, *F*(1,66) = 19.97, *p* < 0.001, ${\eta }_{\rho }^{2}$=0.23, with correct rejection rates lower with higher compared to lower confidence. There was no main effect of transparency, *F*(1,66) = 1.33, *p* = 0.25, and no interaction, *F*(1,66) = 1.22, *p* = 0.27. With low, *t*(66) = 4.27, *p* < 0.001, *d* = 0.47, and high transparency, *t*(66) = 2.81, *p* = 0.01, *d* = 0.31, correct rejection rates decreased with higher compared to lower confidence.

For sensitivity (*d′)*, there was a main effect of transparency, *F*(1,66) = 14.04, *p* < 0.001, ${\eta }_{\rho }^{2}$=0.18, but no main effect of confidence, *F*(1,66) = 1.12, *p* = 0.29, and no interaction, *F*(1,66) = 2.29, *p* = 0.14.

For response bias (*c*), there was a main effect of transparency, *F*(1,66) = 7.31, *p* = 0.01, ${\eta }_{\rho }^{2}$=0.10, and confidence, *F*(1,66) = 58.10, *p* < 0.001, ${\eta }_{\rho }^{2}$=0.47, with greater bias towards agreeing with the automation with higher compared to lower confidence. There was no interaction, *F* < 1. With low, *t*(66) = 7.03, *p* < 0.001, *d* = 1.01, and high transparency, *t*(66) = 5.04, *p* < 0.001, *d* = 0.72, participants were more biased towards agreeing with automation with higher compared to lower confidence.

### Correct decision time

There was main effect of confidence, *F*(1,66) = 60.96, *p* < 0.001, ${\eta }_{\rho }^{2}$=0.48, with faster decision times with higher compared to lower confidence. There was no main effect of transparency, *F*(1,66) = 3.24, *p* = 0.08, and no interaction, *F*(1,66) = 3.53, *p* = 0.07. With low, *t*(66) = 4.98, *p* < 0.001, *d*** = **0.45, and high transparency, *t*(66) = 8.10, *p* < 0.001, *d*** = **0.54, decision times were faster with higher compared to lower confidence.

### Workload

There was a main effect of transparency, *F*(1,66) = 5.49, *p* = 0.02, ${\eta }_{\rho }^{2}$=0.08, and confidence, *F*(1,66) = 25.76, *p* < 0.001, ${\eta }_{\rho }^{2}$=0.28, with lower perceived workload with higher compared to lower confidence. There was an interaction between transparency and confidence, *F*(1,66) = 7.45, *p* = 0.01, ${\eta }_{\rho }^{2}$=0.10. With low, *t*(66) = 4.44, *p* < 0.001, *d* = 0.13, and high transparency, *t*(66) = 4.66, *p* < 0.001, *d* = 0.25, perceived workload was lower with higher compared to lower confidence.

## Discussion

We examined the impact of automation transparency (low, high) and providing automation confidence (somewhat, highly confident) on the accuracy of automation use, decision time, and perceived workload, trust and usability. A core prediction was that transparency and confidence information could have interacting effects on automation use.

### Automation transparency

Increased transparency led to more accurate automation use (improved hit rate and sensitivity), faster correct decisions, lower perceived workload, and increased perceived trust and usability. These findings are in line with prior work reported across a variety of task domains (e.g., air traffic control, robotics, ground troops support, search and rescue, process control) that increased transparency can improve the accuracy of automation use and can have positive effects on other outcome variables (for reviews see Bhaskara et al., 2020; Sargent et al., 2023; Van de Merwe et al., 2022). Compared to the vast majority of studies outlined in these prior reviews of the transparency literature, it is notable that in the current study increased transparency impacted every single outcome variable, although we did not measure situation awareness (Chen et al., 2014). The current study therefore provides strong support for the utility of the automation transparency design principle.

The current transparency findings are also consistent with prior UV management work demonstrating that increased transparency in UV management leads to more accurate automation use (e.g., Mercado et al., 2016; Stowers et al., 2020; Tatasciore & Loft, 2024; Tatasciore et al., 2023), but no difference in correct rejection rates (e.g., Gegoff et al., 2023) and thus a bias towards agreeing with automated advice (e.g., Bhaskara et al., 2021; Tatasciore & Loft, 2024; Tatasciore et al., 2023). Furthermore, the faster correct decision times and lower perceived workload with increased transparency are in line with Gegoff et al., but contrast with prior findings that increased transparency does not induce differences in decision time (Tatasciore et al., 2023) or workload (Tatasciore & Loft, 2024; Tatasciore et al., 2023). Finally, findings of higher perceived trust and usability with increased transparency are consistent with Gegoff et al. (2023) and Tatasciore and Loft (2024), but inconsistent with Tatasciore et al. (2023).

### Automation confidence

There were no benefits to the accuracy of automation use, decision time, workload, or trust and usability ratings for participants provided with automation confidence information. These findings are consistent with prior work that automation confidence, or graded decision aid certainty, did not improve the accuracy of automation use (Bartlett & McCarley, 2017, 2019). In contrast, they are inconsistent with prior findings of positive outcomes of graded likelihood alarms on performance compared with a binary alarm (Wiczorek & Manzey, 2014; Wiczorek et al., 2014). A potential reason for these differences is that in the prior studies reporting benefits to automation use with graded decision aid certainty, participants completed an additional non-automated concurrent task. It might be that with concurrent task demands, and thus potentially less perceived time to verify advice, automation confidence and the associated information expected value cues (Moray & Inagaki, 2000; Wickens et al., 2015) is more depended upon to assist decisions (also in which case high transparency could be particularly useful for the efficient verification of advice). Future research could examine if there are benefits of automation confidence on the accuracy of automation use in UV management tasks with non-automated concurrent task demands (e.g., Tatasciore et al., 2023), as opposed to when a single UV management task aided by automation is completed.

Furthermore, the current findings differ from McGuirl and Sarter (2006) who reported that confidence information (as opposed to overall reliability information) led to lower automation bias in cases of inaccurate automated advice. A key distinction between that study and the current study lies in the experienced reliability levels associated with the reported confidence information. In the current study, higher confidence corresponded to 90% reliability, and lower confidence to 60% reliability. In contrast, in the McGuirl and Sarter study when high confidence was presented, the automation was correct on 89% of trials and when low confidence was presented it was correct on 25% of trials (i.e., worse than chance). With this design, heavily biasing decisions to disagree with the low confidence recommendations would improve response accuracy, and some McGuirl and Sarter participants reported using such a strategy. Future research could seek to systematically examine how participants learn the automation reliability (see Strickland et al., 2024) associated with different levels of automation confidence and subsequently adapt their automation use strategies (Strickland et al., 2021, 2023).

Although there was little overall effect of providing confidence information versus not, within the confidence condition there were clear effects of confidence level from trial-to-trial. Specifically, for trials with low (‘somewhat confident’) compared to high (‘highly confident’) automation confidence, there were decreased hit rates and increased correct rejection rates, consistent with a bias shift against agreeing with advice (i.e., less reliance on automation). Furthermore, and consistent with decreased automation reliance, lower confidence trials were associated with slower decision times and higher perceived workload. Critically, this indicates that participants clearly did attend to, and use, the confidence information provided. It appears that there was little overall effect of confidence condition on outcomes because the high versus low confidence trials approximately balanced each other out (i.e., equal frequency of high vs. low confidence trials). Future research could examine whether with different confidence frequencies (e.g., if “somewhat confident” was rare), providing confidence information could differentially affect overall levels of bias, decision times, and/or perceived workload.

### Interaction between automation transparency and confidence

We hypothesised that transparency and confidence could have interacting effects on automation use, given that low confidence should cue a higher expected value for scrutinising transparency information in moments when automation is perceived to be more likely to error (Moray & Inagaki, 2000; Senders, 1983; Steelman et al., 2011; Wickens et al., 2015) and that if so, higher transparency displays should facilitate subsequent understanding of the rationale underlying advice and what to expect if automated advice is followed (predictability). We also theorised that high transparency may also protect against the negative effect of high confidence signalling on the correct rejection of automated advice (Gegoff et al., 2023; McGuirl & Sarter, 2006). These outcomes would lead to the benefit of increased transparency being greater when automation confidence information was also provided.

We found no evidence of such an interaction between transparency and confidence information. As reviewed above, on low confidence trials there was a shift in bias towards disagreeing with advice, as compared with high confidence trials. Although this benefitted correct rejection rates, there were costs of low confidence information to hit rates, decision times, and perceived workload. Critically, we did not find that these effects were modified by transparency information. Rather, these impacts of low confidence information persisted even in the high transparency condition.

## Limitations and conclusions

The UV task used in this study is broadly representative of environments where operators are provided decision support, but it does not contain the information complexity and multitasking requirements of UV management in the field. Further, novice participants likely have different cognitive skills and motivation than experts. However, studies that have used experts in higher-fidelity tasks [e.g., Pokam et al., 2019 (automated driving supervision); Sadler et al., 2016 (soldier manoeuvring); Skraaning & Jamieson, 2021 (nuclear power plant control); Van de Merwe et al., 2024 (nautical navigation)], have generally reported evidence in support of improved performance with automation transparency.

There are important lessons from these field studies for future research in the laboratory to consider, and to even emulate to the extent possible. First, the use of experts, higher-fidelity simulations, and associated ecologically-valid affordances and constraints make it difficult to neatly operationalise transparency (Skraaning & Jamieson, 2021). Second is that experts are often not responding to controlled/isolated automated advice (proposals), but instead supervising automation to intervene if required, or monitoring automation to enhance situation awareness (e.g., Pokam et al., 2019; Skraaning & Jamieson, 2021). Third, care should be taken when applying transparency to decision time-critical applications such as nautical navigation (Van de Merwe et al., 2024). Fourth, whereas transparency principles can readily be applied to local automation at the component level (as in current study), implementing transparency in *work systems* can change the operational concept (operator roles, task allocation, teamwork), which may produce unexpected effects on system performance (Skraaning & Jamieson, 2021; also see Tatasciore & Loft, 2024).

Another limitation of the current study was that we only used categorical confidence information rather than other forms of confidence information (e.g., numerical). A drawback of categorical confidence is that humans may not be aware of or may misinterpret the threshold criteria of the categories (Bhatt et al., 2021). Future research could manipulate different forms of confidence information (e.g., categorical, numerical).

In conclusion, increased transparency benefited the accuracy of automation use, decision time, and perceived workload, and resulted in higher perceived trust and usability. Although increased transparency can benefit human-automation interaction, designers should be cautious of the fact that increased transparency may also lead humans to over-rely on incorrect automated advice. Automation confidence had no net benefit on the accuracy of automation use. However, there was a bias shift from agreeing with the automated advice when it was highly confident to disagreeing when it was somewhat confident. Thus, confidence information does impact human reliance on automation, which is problematic in the rare occurrence that confidence information is incorrect (i.e., if automation has missed or does not have access to certain information). Increased transparency did not influence the effects associated with this shift in bias, and thus did not interact with confidence information to further improve the accuracy of automation use. That said, we cannot rule out the fact that in field settings (e.g., where there may be concurrent task demands and other affordances/constraints) transparency and confidence may interact to further benefit human-automation interaction.

## Data Availability

The data used and/or analysed during the current study are available from the corresponding authors on reasonable request.

## Abbreviations

ATWIT: Air traffic workload input technique
SAT: Situation-awareness agent-based transparency
SUS: System usability scale
UV: Uninhabited vehicle
UAV: Uninhabited aerial vehicle
UGV: Uninhabited ground vehicle
USV: Uninhabited surface vehicle
