# Correction: Transparency improves the accuracy of automation use, but automation confidence information does not

**DOI:** 10.1186/s41235-025-00631-8

**Published:** 2025-05-11

**Authors:** Monica Tatasciore, Luke Strickland, Shayne Loft

**Affiliations:** 1 The University of Western Australia, 35 Stirling Highway, Perth, WA 6009 Australia; 2 Curtin University, Perth, Australia

**Correction: Cognitive Research: Principles and Implications (2024) 9:67** 10.1186/s41235-024-00599-x

The authors wish to note in Table 3 of the original article that ‘0.86 (0.08)’ should instead be attributed to Low T, and ‘0.91 (0.07)’ should instead be attributed to High T.

